# Bioactive Molecules of Mandarin Seed Oils Diminish Mycotoxin and the Existence of Fungi

**DOI:** 10.3390/molecules26237130

**Published:** 2021-11-25

**Authors:** Salman S. Alharthi, Ahmed Noah Badr, Karolina Gromadzka, Kinga Stuper-Szablewska, Adel Gabr Abdel-Razek, Khaled Selim

**Affiliations:** 1 Chemistry Department, College of Science, Taif University, P.O. Box 11099, Taif 21944, Saudi Arabia; s.a.alharthi@tu.edu.sa; 2 Food Toxicology and Contaminants Department, National Research Centre, Dokki, Cairo 12622, Egypt; 3 Chemistry Department, Poznan University of Life Science, ul. Wojska Polskiego 75, 60-625 Poznań, Poland; karolina.gromadzka@up.poznan.pl (K.G.); kinga.stuper@up.poznan.pl (K.S.-S.); 4 Fats and Oils Department, National Research Centre, Dokki, Cairo 12622, Egypt; adelgabr2@gmail.com; 5 Food Science and Technology Department, Faculty of Agriculture, Fayoum University, Fayoum 6351, Egypt; kas00@fayoum.edu.eg

**Keywords:** aflatoxin B_1_, antimicrobial, biomolecules, biodegradation, mandarin seed oil, mycotoxin, oil mix, toxigenic fungi

## Abstract

Mandarin is a favorite fruit of the citrus family. Mandarin seeds are considered a source of nontraditional oil obtained from byproduct materials. This investigation aimed to assess the biomolecules of mandarin seeds and evaluated their antimycotic and antimycotoxigenic impact on fungi. Moreover, it evaluated the protective role of mandarin oil against aflatoxin toxicity in cell lines. The two types of extracted oil (fixed and volatile) were ecofriendly. The fatty acid composition, tocopherol, sterols, and carotenoids were determined in the fixed oil, whereas volatiles and phenolics were estimated in the essential oil. A mixture of the two oils was prepared and evaluated for its antimicrobial impact. The reduction effect of this mixture was also investigated to reduce mycotoxin secretion using a simulated experiment. The protective effect of the oil was evaluated using healthy strains of cell lines. Fixed oil was distinguished by the omega fatty acid content (76.24%), lutein was the major carotenoid (504.3 mg/100 g) and it had a high *β*-sitosterol content (294.6 mg/100 g). Essential oil contained limonene (66.05%), *α*-pinene (6.82%), *β*-pinene (4.32%), and *γ*-terpinene (12.31%) in significant amounts, while gallic acid and catechol were recorded as the dominant phenolics. Evaluation of the oil mix for antimicrobial potency reflected a considerable impact against pathogenic bacteria and toxigenic fungi. By its application to the fungal media, this oil mix possessed a capacity for reducing mycotoxin secretion. The oil mix was also shown to have a low cytotoxic effect against healthy strains of cell lines and had potency in reducing the mortality impact of aflatoxin B_1_ applied to cell lines. These results recommend further study to involve this oil in food safety applications.

## 1. Introduction

Mandarin (*Citrus sinensis*) belongs to the citrus fruit group, which is characterized as containing numerous vitamins or minerals and is preferrably consumed in winter [1]. Mandarin is recognized as a fruit beloved by the greatest portion of society, where the acidic taste is not felt as much as for others, such as oranges and lemons [2]. The seeds are one of the byproducts that result from fruit consumption, which does not take into account the importance of minor component contents, and they are disposed of in the waste [3]. Mandarin seeds contain a fixed oil besides the presence of an essential oil [4]. The fixed oil regularly consists of fatty acids, and sometimes it contains the oxidizing form of fatty acids. Oxylipins are components that occur due to the oxidation of polyunsaturated fatty acids [5]. These components are known to have antimicrobial effects, particularly against fungi. Moreover, fatty acids such as palmitic acid have been recognized to have bioactivity against microorganisms [6].

Mandarin seeds, as other plant seeds, are rich in minor components. These components act as the shield that protects the seed against spoilage and damage [7]. Bioactive components include several constituents such as oligo- and polysaccharides, dietary fibers, phenolics, flavonoids, tocopherol, sterols, and carotenoids [8]. As the seed is considered the new generation of the plant, the existence of these molecules provides security and safety support for the germ growth of the newborn plant [9]. Moreover, most of these components participate in the antioxidant potency of the obtained extract [10]. The antioxidant activity is significant for avoiding the harmful impact of oxidative stress, which is connected to diseases resulting from the existence of free radicals in the growth environment of seeds [11].

Furthermore, there is a large category of harmful microorganisms known as toxigenic fungi. These fungi possess the capacity to produce chemical substances recognized as mycotoxins [12,13]. These toxins are secondary metabolites secreted by fungi, and as highly dangerous compounds they act as a hidden enemy threat concerning crop production, as well as, animals and public health. Several types of mycotoxin are classified as precarcinogenic chemical compounds, with mutagenic, teratogenic, and immune suppressive effects [14]. Aflatoxins, as well as, ochratoxins are known to cause harmful changes for living cells in experimental animal tissues [15]. Generally, toxigenic fungi are known to produce mycotoxin due to their exposure to an oxidative stress impact [16]. The enrichment of the environment where fungi grow may redirect fungal spores to vegetative growth instead of secondary metabolite production [17]. However, plant extracts were reported with a protective impact against mycotoxin toxicity in experimental animal tissues [18].

The application of plant extracts as a rich source of bioactive molecules in the growth media of fungi could affect their metabolic pathways [13]. These changes decrease the harmful impact of fungal contamination due to the absence or by limiting the existence of secondary metabolites [19]. The present investigation aimed to determine the antimycotic and antimycotoxigenic impact of bioactive molecules that are present in mandarin seed oils. The mixture of essential and fixed oils obtained from the seeds was evaluated for the content of bioactive molecules. The mixture was applied to the microbial media of pathogenic bacteria and toxigenic fungi to estimate its capacity as an antibacterial and antifungal agent. Moreover, the antimycotoxigenic effect was determined using simulated growth media as a means to reduce mycotoxin secretion.

## 2. Materials and Methods

### 2.1. Chemicals and Materials

Chemicals utilized in the present investigation included fatty acid methyl esters (37 FAME mix), tocopherol (*α-,* *β-, γ-,* *δ*-) (≥99% purity), aflatoxin B_1_, ochratoxin A, ochratoxin B, aflatoxin G_1_, and microbial media for microorganism cultivation and evaluations. These were obtained from Sigma-Aldrich Chemical Co. (St. Louis, MO, USA). The standard of all applied chemicals and solvents were of analytical or chromatographic grade.

The antibacterial assay was performed against four Gram-positive bacteria, *Bacillus cereus* EMCC 1080, *Streptomyces avermitilis* ATCC 31267, *Micrococcus luteus* ATCC 15176, and *Staphylococcus aureus* ATCC 13565, and four Gram-negative bacteria, *Escherichia coli* O157-H7 ATCC 51659, *Salmonella typhi* ATCC 15566, *Pseudomonas aeruginosa* NRRL B-272, and *Klebsiella pneumonia* LMD 7726. These strains were obtained from the DSMZ microbial collection (Leibniz Institute DSMZ-German Collection of Microorganisms and Cell Cultures, Braunschweig, Germany), cultured on nutrient agar slants at 37 °C for 24 h, and stored in the refrigerator at 4 °C until use.

The antifungal assay was performed against six strains of toxigenic fungi, *Aspergillus flavus* ITEM 698, *Aspergillus parasiticus* ITEM 11, *Aspergillus carbonarius* ITEM 5010, *Aspergillus ochraceous* ITEM 282, *Penicillium verrucosum* NRRL 965, and *Penicillium chrysogenum* ATCC 10106, obtained from the agro-food microbial culture collection (ITEM), ISPA, CNR, Italy. Fungal strains were stored on Czapek-Dox media before the evaluation test.

### 2.2. Hydrodistillation of Seeds’ Volatile Content

The hydrodistillation was performed for up to 4 h in a circulatory Clevenger-type apparatus up to the exhaustion of the volatiles contained in the mandarin seeds. The Clevenger-type of hydrodistillation extraction of the volatile components was performed according to the British Pharmacopoeia [20] using 2 L Clevenger-type hydrodistillation units. The samples of air-dried seeds (100 g) were charged to the unit, and the yield of extracted volatiles ranged between 1.02 and 1.09 g/100 g of extracted seeds.

### 2.3. Oil Extraction

Fresh mandarin seeds (5 kg) were obtained from the fruits and placed in a manual hydraulic press with perforated stainless steel trays (200 kg/cm^2^; 30 cm diameter × 15 stacked trays). The oil was liquefied in a collection basin after the seeds were loaded onto trays. To obtain crude oil, a hydraulic pressure of 150 kg/cm^2^ was applied for 15 min. The collected oil (534 mL oil) was kept in an amber bottle until it was used again.

### 2.4. Estimation for Mandarin Fatty Acids in the Seed Oil

The fatty acid methyl esters (FAMEs) were made using the AOCS Official Method Ce 1k07 as described previously [19]. At a 1.5 mL H₂/min flow rate, diluted FAMEs were separated on an HP 5890 series II (Hewlett-Packard, Palo Alto, CA, USA) equipped with an FID and Innowax column (30 mm × 0.20 mm × 0.20 µm). The column and detector isotherm temperatures were set to 210 °C and 240 °C, respectively. The fatty acids were identified by comparing retention periods to verified standards.

### 2.5. Determination of Tocopherol Content for Mandarin Oil 

An HPLC Agilent 1100 system (Agilent Technologies, Hewlett-Packard Strasse 876337 Waldbronn, Germany) was used to determine Tocopherol content [21]. At a flow rate of 1.5 mL/min, isocratic methanol was used as the mobile phase. An Extend-C18, Zorbax column (250 mm × 4.6mm × 5 µm, Agilent Co.) was used for chromatographic separation. The UV detector was set to 292 nm and the column temperature was set to 40 °C. The mandarin oil was diluted, and 20 µL was injected, with the measured values integrated and recorded using a Hewlett-Packard Chem-station program Manager.

### 2.6. Carotenoid and Sterol Content Determination

According to procedures that were described by Stuper-Szablewska et al. [22], sterol content was determined. Sterol analysis was done using an Aquity H class UPLC system equipped with a Waters Acquity photodiode array detector (Waters, Milford, MA, USA). Chromatographic separation was achieved on an Acquity UPLC^®^ BEH C18 column (100 mm × 2.1 mm × 1.7 μm) (Waters, Ireland). The elution was isocratically performed using a mobile phase of: A, acetonitrile 10%; B, methanol 85%; C, water 5%, and flow of 0.5 mL/min. The injection volume was 10 µL. Sterol concentrations were measured using an external standard at wavelengths λ = 210 nm (desmosterol, cholesterol, lanosterol, stigmasterol, *β*-sitosterol) and λ = 282 nm (ergosterol). Compounds were identified by comparing the retention times of the investigated peak and the reference, as well as by adding a particular quantity of the standard to the tested sample and repeating the tests. The detection limit was 1 mg/kg. Carotenoids were evaluated by utilizing Acquity ultra-high performance liquid chromatography (Waters, Milford, MA, USA) as described by Kurasiak-Popowska et al. [23].

### 2.7. Phenolic Compound Extraction from Mandarin Oil 

The phenolic content of the oil was extracted using a modified methodology of Ramadan et al. [24]. A quantity of the oil (1 g) was diluted in hexane (3 mL), subsequently, aqueous methanol (5 mL; 80%) was added then shaken well (3 min/vortex). After a minute of repose, the tube was centrifuged (4200 g/10 min), and the hydro-alcoholic layer was separated using a Pasteur pipette. The extraction procedure was conducted twice more, and the results were collated. The polar extract was dissolved in 10 mL acetonitrile then washed repeatedly with hexane. The purified extract was vacuum-evaporated to near dryness in a rotary evaporator (35 °C) before being dissolved in aqueous methanol. The final extract was utilized for further determinations.

### 2.8. Determination of Phenolic and Flavonoid Fractions

The phenolic fraction constituents in the previously prepared extract of mandarin seeds were determined using the technique established by Stuper-Szablewska et al. [22]. The quantities of phenolic content were measured at 320 and 280 nm using retention time comparisons of analyte peaks with the retention time for references and by introducing a particular quantity of the reference to the examined extract and performing the assay. The limit of quantification was 10 ng/g sample, and the results were calculated in triplicate and given in means ± SEM.

### 2.9. Determination of Mandarin Seeds’ Volatile Content

Measurements were performed on an Agilent 7890A GC system with a split injector port set to 200 °C and a 200:1 split ratio. The Agilent 7697A autosampler was used for static headspace sampling. Vials were equilibrated at 70 °C for 10 min before sampling with a 50 L loop (85 °C). The transfer line input temperature was kept constant at 95 °C and separation was accomplished with a DB-624 capillary column (30 m × 0.25 mm I.D. 1.4-m film thickness) with a constant helium carrier flow of 1 mL/min. The initial oven temperature was 45 °C for 3 min, followed by a 25 °C/min ramp to 240 °C for 3 min. The Agilent detector 5975 MSD was engaged in the scan range (29–250 *m*/*z*; rate of 6.1 scans/s) to identify and quantify volatile compounds. All calibration curves and sample concentrations were calculated using (B.09.00) Mass-Hunter software. 

### 2.10. Determination of Antimicrobial Activity

The activity of oil samples was evaluated by the methodology described previously [19]. We evaluated the inhibitory effect of the mandarin oil against pathogenic bacteria (Gram-positive and Gram-negative strains), and versus toxigenic fungi using agar diffusion techniques [25]. The samples were diluted with phosphate buffered saline (PBS) at a pH equal to 7.1 ± 0.1. The same number of subsequent concentrations were performed. The minimal concentration of the samples that inhibited microbial growth was determined using the microdilution method by serially diluting in sterile nutrient broth for bacteria and Czapek-Dox broth for fungi [26].

### 2.11. Simulation Experiment for the Evaluation of Mycotoxin Inhibition 

A volume of Czapek-Dox medium equal to 150 mL was autoclaved in 500 mL conical flasks for the assessment of the influence of mandarin oil samples on fungal mycotoxin formation. In flasks, two strains (*A. parasiticus* and *A. carbonarius*) were grown separately with and without the application of oil in growth media. The strain of *A. parasiticus* fungi is known to produce aflatoxins, whereas the strain of *A. carbonarius* fungi is known to secrete ochratoxins. The toxin concentrations of the medium filtrates after incubation (26 °C/6 days) were also determined using the HPLC system, as described by Shehata et al. [27] for aflatoxin determination and Badr et al. [28] for ochratoxin determination.

### 2.12. Determination of Mycotoxin

An Agilent 1100 (Agilent Technologies, Hewlett-Packard Strasse 876,337 Waldbronn, Germany) high-performance liquid chromatography system was utilized to determine mycotoxin concentrations in the investigated samples. An Extend-C18, Zorbax column (250 mm × 5 µm, Agilent Co.) was used for chromatographic separation. The column temperature was set to 40 °C, and the flow rate was set to 1.0 mL/min; the injection volume for the samples and standard was set to 10 µL. The detector was set to excite at 360 nm and emit at 440 nm for aflatoxins, and it was set to excite at 330 nm and emit at 450 nm, respectively for ochratoxins. Data were integrated and recorded using a Hewlett-Packard Chem-Station program Manager.

### 2.13. Prevention Effect of Mandarin Oil against Mycotoxin Cytotoxicity 

The normal hepatic cell line strains of HL-7702 and AML12 were cultivated at a density of 1 × 10^4^ cells/well (100 µL) in DMEM culture medium supplemented with antibiotics (20 mg of amoxicillin and 25 mg chloramphenicol in 0.9% saline), 10% phosphate buffered saline (PBS), and incubated 24 h/ 37 °C/ 5% CO_2_ [29]. After attachment, a serially diluted extract was administered at doses ranging from 1000 to 6.25 g/mL for AML12 and HL-7702 cells. Following that, 10 µL of a 12 mM MTT stock solution (5 mg/mL MTT in sterile PBS) was applied to each well. The MTT solution was removed after 4 h of incubation at 37 °C. The change in the percentage of surviving cells against the applied concentrations was utilized to demonstrate the curve according to equation (1) as described by Liu et al. [30].

% of surviving cells = [(ODs − ODb)/(ODc − ODb) × 100%](1)

where,

ODs: optical density of sample evaluated.
ODb: optical density of blank evaluated.
ODc: optical density of the control evaluated.

AFB_1_, which is deemed as a toxic compound causing decreases in cell viability for the normal tested cell line, was applied as a positive control. In the same experiment, the mandarin oil influence that reduced the AFB_1_ toxicity was also evaluated.

### 2.14. Statistical Analysis

The results were expressed as mean values ± standard error of means (SEM) calculated from three replicates. The statistical analyses of data and graphs were performed using ANOVA tests and were expressed using GraphPad Prism 7 (GraphPad Software Inc., San Diego, CA, USA).

## 3. Results

### 3.1. Mandarin Fatty Acid Composition in Seed Oil

Fatty acid compositions of mandarin seed oil are given in Table 1, where the other significant calculated values were also registered in the second part of the table.

Linoleic acid (omega 6; 40.55%) was established as the dominant fatty acid in mandarin seed oil, followed by oleic (22.99%), myristoleic (22.9%), linolenic (omega-3, 6.13%), and palmitoleic (6.21%). At the same time, mandarin seed oil contained the following total fatty acid groups: monounsaturated fatty acids (MUFA 52.15%), polyunsaturated fatty acids (PUFA 46.99%), and saturated fatty acids (SFA 0.78%). The fatty acid group balance (SFA:MUFA:PUFA) was recorded as 0.02:1.56:1.41 in mandarin seed oils.

It is significant to point out the existence of omega fatty acids in this oil, where the results recorded ω-3, ω-6, ω-7, and ω-9 fatty acids. The majority of the omega content was joined to the ω-3 fatty acids, followed by ω-7 and ω-6 fatty acids. However, more than one fatty acid is considered to belong to ω-9, but their sum is recorded as the lowest content. Again, the ratio between the ω-6 and ω-3 content recorded is not compatible as referred to by global nutritional organizations.

### 3.2. Carotenoid and Sterol Fractions of the Mandarin Oil

The oil content of the carotenoid and sterol fractions of mandarin seeds is represented in Table 2; eight fractions of carotenoids were recorded to exist in the analyzed sample of oil. It was distinguished by the higher content of the lutein fraction as the dominant carotenoid, followed by xanthine. Phytoene and *α*-carotene were recorded in traces. Moreover, five sterol fractions were determined as the majority for the *β*-sitosterol fraction, while the campesterol fraction came in as having the second-highest sterol content of the mandarin oil.

### 3.3. Mandarin Oil Content of Tocopherol

The tocopherol content of mandarin seed oils is presented in Table 2. The total content of tocopherol is 42.38 mg/100 g for mandarin seed oils. The samples were characterized by a much higher percentage of *α*- and *γ*-tocopherols. The content of *α*-tocopherol was the major fraction in seed extract and it reached 64.74%, while the second most abundant component was *γ*-tocopherol represented by 14.85 mg/100 g (35.04%), *β*-tocopherol was recorded as not detectable, while *δ*-tocopherol fractions appeared in traces.

### 3.4. Phenolic and Flavonoid Fractions of Mandarin 

Regarding the results represented in Table 3, the mandarin seed oil is shown with a significant content of phenolic compounds like phenolic acids and flavonoids. Out of ten phenolic acids, the major content was recorded for gallic acid, followed by syringic acid and *trans*-ferulic acid. Moreover, the lowest content of the existing phenolic acids was found for vanillic acid. Flavonoids in mandarin seed oil were also determined, the results pointed out catechol was the dominant flavonoid compound of mandarin oil. The oil content of catechin and isorhamnetin were recorded as somewhere close in their amounts. The lowest flavonoid content was shown for hesperidin, then epicatechin. Two flavonoid compounds were recorded as not detected, namely luteolin and chrysin, while the result reflected a significant content of quercetin.

### 3.5. Volatile Content of Mandarin Seeds 

Concerning the volatile content, which was obtained using the hydrodistillation technique, it was reported by the presence of twenty-two compounds named in Table 4**.** Most of the recorded percentages for the seeds’ essential compound content are represented by very low values. Notwithstanding, limonene was reported as having the major percentage of the essential compounds, it was shown as the dominant compound of the volatile content for mandarin seeds. The compound of *γ*-terpinene was determined in the volatile content of mandarin seeds at about 12% of the total volatile content. It is worth mentioning that, *α*-pinene and *β*-pinene existed in the volatile content with considerable percentile ratios. The compound, namely decanal, which is considered an aldehyde of a saturated fatty acid (capric acid) was represented by the lowest percentile ratio of mandarin seed content of the volatile compounds.

### 3.6. Antimicrobial Activity of Mandarin Seed Oil

**Figure 1:**
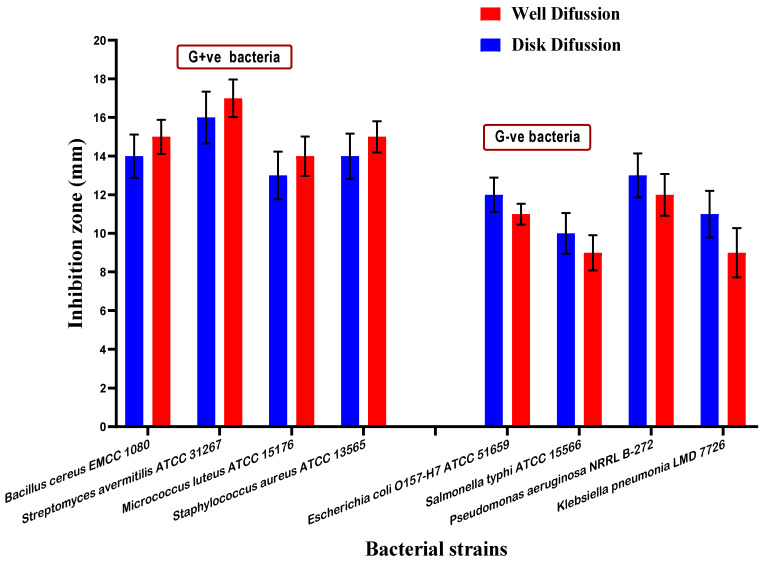
The antibacterial activity of mandarin seed oil against pathogenic strains of bacteria. G+ve bacteria represent the oil activity against the Gram-positive strains. G−ve bacteria represent the oil activity against the Gram-negative strains.

From this point forward, the expression, seed oil, will refer to the mixed oil (fixed and volatile) extracted from mandarin seeds (MMOS). The antimicrobial activity of mandarin mixed oil of seeds (MMOS) was determined. At the first stage, MMOS was evaluated against pathogenic strains of bacteria. The bacterial strains were classified as four Gram-positive strains and four Gram-negative ones. The experiment was performed using two diffusion assays (disk, and well) to ensure the results’ accuracy. The zones of inhibition for the bacterial growth indicate a good efficiency of MMOS to reduce bacterial growth (Figure 1), which is considered a significant indication of its antibacterial impact. In this regard, the results could refer to MMOS as a bioactive extract that may participate in shelf-life extension.

However, the second part of the evaluation for MMOS was performed with regard to the antifungal impact. The seed oil was applied in fungal media using the same assays as for bacteria. The toxigenic strains of fungi which are known to produce mycotoxins, are more dangerous and were targeted for the application of MMOS for the evaluation of antifungal activity.

**Figure 2:**
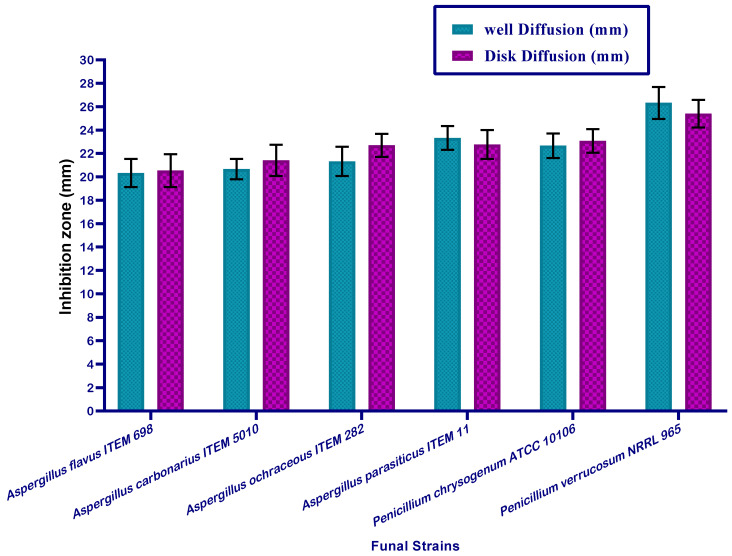
Antifungal activity recorded for mandarin oil versus toxigenic strains of fungi.

The results represented in Figure 2 reflect a similar impact of the application on the strains of *Aspergillus* fungi. The evaluated strains of *Aspergillus* fungi can produce aflatoxin and ochratoxin types of mycotoxins. MMOS as a plant extract contains a variable content of biomolecules, and the impacts could be synergistic with each other in order to express antifungal effects and this could be important when describing the inhibition of fungal growth. The values of the inhibition zones, which were recorded for the tested strains of toxigenic fungi were shown by a considerable impact. This point is significant with regard to the application of this oil to provide safety properties against fungal infection in stored products of food and feed. 

### 3.7. Mycotoxin Inhibition Simulation

After the evaluation of MMOS as an antifungal in the previous experiment, two strains of the toxigenic fungi were selected for the evaluation in liquid media. In this step, the fungal growth occurred in Czapek-Dox broth media to record the impact of MMOS using a simulated experiment. The simulation was done against *A. parasiticus* that is known to produce aflatoxins (AFB_1_ and AFG_1_) in the liquid growth media. Moreover, it was applied in the media of *A. carbonarius*, where the inhibition of mycelia growth for the the two tested strains was evaluated in control flasks, flasks treated by the volatile oil, flasks treated by the fixed oil, and flasks treated by the MMOS extract. The results that represent a reduction of the toxigenic fungal growth of these two *Aspergillus* fungi are represented in Figure S1. 

**Figure 3:**
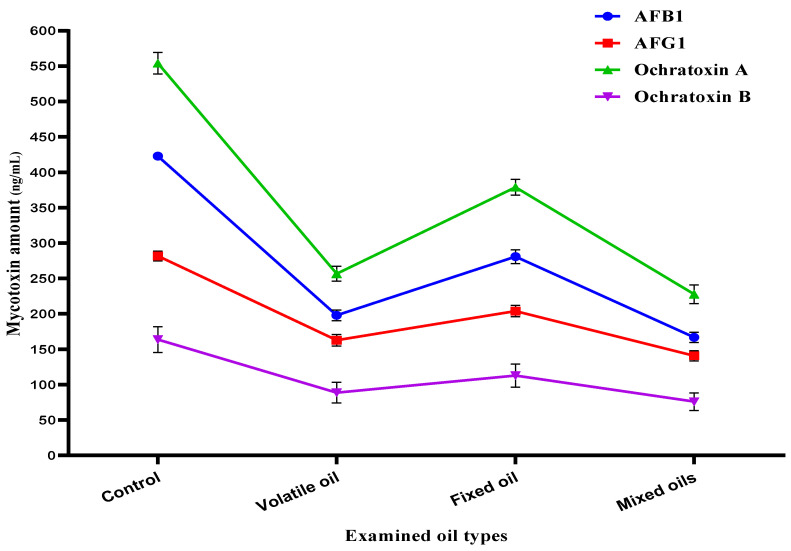
Antimycotoxigenic potency of the mandarin oil types obtained from seeds. AFB_1_: aflatoxin B_1_; AFG_1_: aflatoxin G_1_.

The liquid media of the fungi filtrates were collected individually for mycotoxin evaluations. The media utilized for the strain of *A. parasiticus* was targeted to determine the reduction expected for aflatoxins (AFB_1_ and AFG_1_), while the media utilized for the *A. carbonarius* fungal strain was targeted to determine the reduction expected for ochratoxins (OCA and OCB). According to the results that are shown in Figure 3, the volatile oil was more effective in reducing the mycotoxin secretion by the applied strains of fungi. Moreover, where the mixed oil of MMOS was present in the growth media, a little more reduction of aflatoxin and ochratoxin secretion was shown in the estimated mycotoxin content in the liquid growth media. 

### 3.8. Prevention Effect of MMOS against Mycotoxin Cytotoxicity

**Figure 4:**
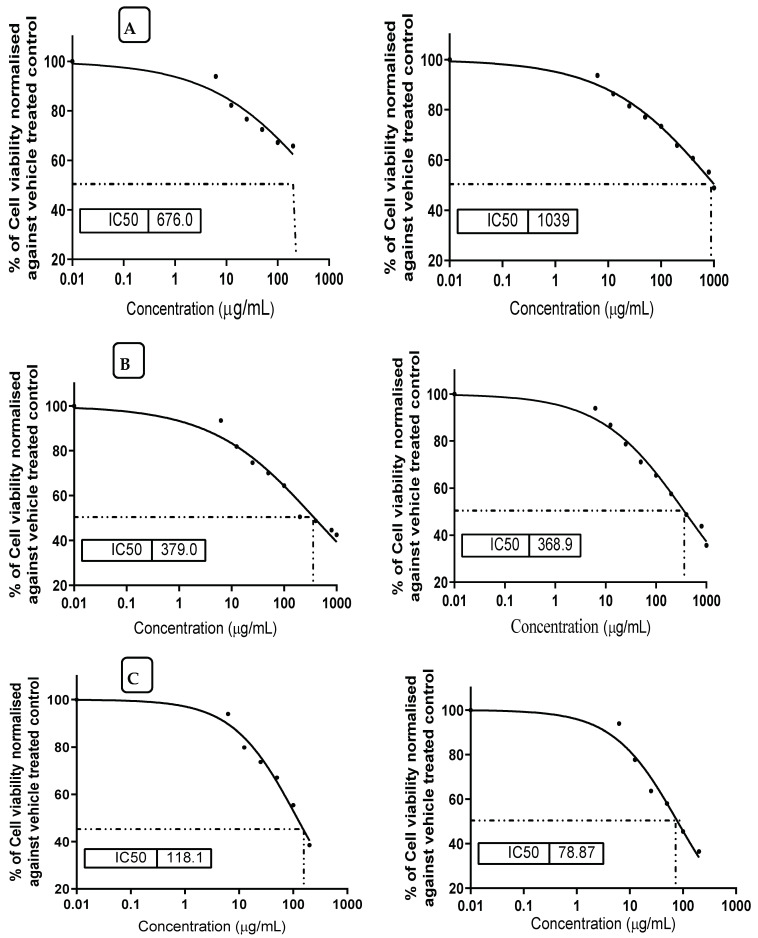
Values of IC**_50_** for HL-7702 (left) and AML12 (right) of healthy cell lines against aflatoxin B_1_ and the mix of mandarin seed oil micelles. (**A**) cell line of HL-7702 (left) and AML12 (right) treated by mandarin oil. (**B**) cell line of HL-7702 (left) and AML12 (right) treated by mandarin oil in the presence of aflatoxin B_1_. (**C**) cell line of HL-7702 (left) and AML12 (right) treated by aflatoxin B_1_.

The impact of mandarin seed oil on the viability of healthy cell lines, where aflatoxin B_1_ was present or absent in culture media, was determined. Again, the impact of aflatoxin B_1_ alone was evaluated against the tested cell lines of the two types. It was recorded that the application of mandarin micelles individually showed high IC**_50_** values for the HL-7702 or the AML12 cells, indicating a better safety indication of this oil on its application (Figure 4A). However, aflatoxin B_1_ represented a cytotoxic impact when it was applied in the growth media of the cell lines (Figure 4C). This impact was decreased as an acute effect when mandarin oil was implemented in the growth media (Figure 4B). Referring to the results of cell viability, the values recorded for the IC**_50_** for the HL-7702 or AML12 cells were close when grown in the presence of mandarin oil in media with AFB_1_ present. 

Regarding the result of aflatoxin toxicity on the two types of cell lines, it was shown by an inhibition concentration, IC**_50_**, of around 100 AFB_1_/mL in the cell line growth media. These values were considered acute doses, which is reflected by the high mortality of the cell line viability. On the other hand, the application of mandarin micelles in the growth media of cell lines that contained AFB_1_ as a cytotoxic compound, showed a high performance, leading to cell viability being recovered even in the presence of the toxic compound.

Notwithstanding, mandarin oil, which consists of volatile and nonvolatile components, represents safe characteristics due to the behavior of the oil recorded against the viability of the two examined strains of healthy cell lines (HL-7702, and AML12). 

## 4. Discussion

Plant extracts, particularly nontraditional ones, contain various biomolecules and active components. These components have been recorded to have the capacity to reduce the harmful impact of hazardous compounds that exist in the plant growth environment [31]. The contents of the seeds of plants normally possess several minor components, which serve in their defense system to protect the second plant generation [32]. While plant germination may suffer from unsuitable environmental conditions during growth, the existence of these minor components can provide protection throughout the early stage of germination. Generally, these unsuitable conditions are linked to oxidative stress occurring due to the presence of reactive oxygen species (ROS) and free radicals. The existence of biomolecules that possess an antioxidant potency may have a vital function in suppressing the occurrence of oxidative stress, which leads to providing a protective system against microbial infections. The issue could be solved through the natural antioxidants from plant extracts [33].

Mandarin seeds are one of the citrus plants’ germs, in which growth will result in newborn mandarin trees. The analysis of these seeds by evaluating their content of fatty acids show a significant content of omega fatty acids, where oleic and palmitic acids derivatives are present. Palmitic as well palmitoleic acid are known to have an antimicrobial impact [10,34]. This impact was clearly shown by the evaluation of oil against bacterial (Figure 2) and fungal strains (Figure 3) throughout the antimicrobial evaluation of the oil. Again, the oil content of polyunsaturated fatty acid (PUFA) was recorded as close to half the content of the fatty acid composition of mandarin seed oil. The PUFAs are generally known to possess antimicrobial effects, particularly against bacterial strains of microorganisms [35]. Moreover, it was pointed out that fatty acids that are classified as ω-3 fatty acids sourced from plants possess activity as antibacterial agents [34].

Other minor molecules belonging to carotenoids and sterols, and tocopherol were recorded to be present in the analyzed sample of the mandarin seed oil. Lutein, which was recorded as the dominant carotenoid, has been reported to possess high antioxidant activity and anti-inflammatory potency [36]. This antioxidant activity acts in the antimicrobial impact. For the sterols, the *β*-sitosterol fraction was recorded as the major component of the sterol content (Table 2). However, this fraction is known to possess antimicrobial potency, particularly regarding fungi [37]. The oil content of tocopherol was distinguished by the presence of *α-* and *γ*-fractions, the absence of a *β*-fraction and a *δ*-fraction recorded as traces. The *α*-tocopherol fraction was reported to possess antibacterial activity [38], whereas other investigations have referred to *α-* and *γ*- tocopherol fractions with antifungal impact [39]. The existence of fatty acids, tocopherol fractions, and phenolic compounds was reported to enhance the antimicrobial characteristics of plant extract [40].

The phenolic content of mandarin seeds (Table 3) participated in the antifungal impact that was shown against the evaluated toxigenic strains of fungi recorded in Figure 2, as well as their impact against the pathogenic bacterial strains shown in Figure 1. The phenolic content of plant extract was reported previously to cause a reduction of fungal growth [31]. The presence of phenolics, flavonoids, and minor components in the extract content leads to more inhibition of fungal contamination [41]. Moreover, the impact of phenolic content is extended to reduce the amount of toxin secretion (Figure 3) that can occur in the media from fungal growth [42]. 

The presence of essential and volatile compounds in the oil mix (MMOS), which is formed by blending the two mandarin oils, participates in its antimicrobial potency. It was recorded that limonene was the dominant volatile fraction. Limonene was reported as having an antibacterial effect against numerous strains of bacteria [43]. The antifungal activity of limonene was also recorded, particularly against the toxigenic fungal strain of *Fusarium* [44]. 

The volatile content of *γ*-terpinene was shown to be high, and it has the capacity to reduce fungal growth in contained media [45]. Moreover, the fractions of *α*- and *β*-pinene existed in considerable amounts in mandarin seeds. These molecules are known for their antibacterial and antifungal effects [46]. However, *α*-pinene and *β*-pinene were reported to have a considerable antifungal impact [47]. This impact may elucidate the better reduction of toxigenic fungal growth and the inhibition that occurred with the presence of MMOS in the toxigenic growth media. It was previously reported that the existence of volatile molecules in the applied oil, was reflected by a great antifungal potency in [45]. 

Commonly, aflatoxins are known as pre-carcinogenic compounds that lead to hepatocellular carcinoma, mutagenic, and teratogenic changes in biological tissues [15]. So, their existence in the growth media of healthy strains of cell lines could suppress their growth and reduce cell viability [19]. Aflatoxins are classified as free radicals [48], where their presence in the biological cell growth cycle and in the control of cell metabolism systems gives rise to damage [18]. 

Plant molecules, which are recognized by their antioxidant potency act to inhibit the oxidative stress of aflatoxins that exist within the biological system [49]. Where MMOS is recorded as having a significant content of bioactive molecules it possesses efficacy to serve as an antioxidant. The implementation of mandarin seed oil in the media with toxigenic fungal growth reduced the oxidative stress of unsuitable conditions (Figure 2 and Figure S1).

This stress is considered the main reason forcing the fungi to secrete mycotoxin [17,48]. Moreover, the existence of MMOS in the media utilized for cell viability evaluations was shown by its activity to decrease the mortality impact of aflatoxin B_1_ when they existed together in the cell line media. This impact may also be linked to the impact of the oil itself in the cell line media [19]. The novelty of this work is connected to the characterization of a nontraditional type of extract with the capacity to fight the contamination of toxigenic fungi and lead to a reduction of mycotoxin secretion. This extract will provide a new approach to make crops and food materials safe against spoilage and is nonharmful, which gives a wide scope for consumption in a safe form.

## 5. Conclusions

Mandarin, like other fruits and vegetables have plant byproducts that are rich in biomolecules. Mandarin seeds contain various minor components in their oil, including omega fatty acids, carotenoids, sterols, and tocopherol. These contents are known to have potency, particularly as antimicrobial agents. Phenolics and flavonoids are the minor components that possess antioxidant activity. The great hazard facing food safety and that threatens plant security is mycotoxin. These chemical substances are secreted by toxigenic fungi and can lead to the occurrence of biological issues in the biological system. The examination of MMOS to inhibit toxigenic fungal growth, reduce mycotoxin secretion, and increase the viability of cell lines treated by aflatoxin B_1_ was investigated. The results reflected a significant impact that indicates the efficiency of MMOS to protect cell line viability against the mortality effect of aflatoxin, and inhibit the fungal growth of applied toxigenic fungi; as well as the activity of minor content components of the oil that possess antioxidant potency. This antioxidant activity is recorded by the capability of fighting the impact of oxidative stress, leading to a more protective effect. These recommend the application of oil for the preservation of food material as a novel approach, which will be able to fight toxigenic fungal contamination and their metabolites. 

## Figures and Tables

**Table 1:** Fatty acid composition of mandarin seed oil.

Carbon Number	Fatty Acids	Concentration (%)
**C12:0**	**Lauric**	0.08 ± 0.001
**C14:0**	**Mayristic**	0.02 ± 0.001
**C14:1**	**Myristoleic**	22.9 ± 0.96
**C16:0**	**Palmetic**	0.51 ± 0.09
**C16:1 ω-7**	**Palmitoleic**	6.21 ± 0.22
**C18:0**	**Stearic**	0.03 ± 0.005
**C18:1 ω-6**	**Oleic**	7.87 ± 0.67
**C18:1 ω-9**	**Oleic**	15.12 ± 0.97
**C18:2 ω-3**	**Linoleic**	40.55 ± 1.08
**C18:3 ω-6**	**Linolenic**	6.13 ± 0.34
**C20:0**	**Arachidic**	0.06 ± 0.002
**C20:1 ω-9**	**Gadoleic**	0.04 ± 0.004
**C20:2 ω-6**	**Eicosadienoic**	0.02 ± 0.006
**C20:3 ω-9**	**Mead**	0.17 ± 0.02
**C20:4 ω-6**	**Arachidonic**	0.09 ± 0.003
**C20:5 ω-3**	**Eicosapentanoic**	0.01 ± 0.002
**C22:0**	**Behenic**	0.03 ± 0.005
**C22:1 ω-9**	**Erucic**	0.01 ± 0.001
**C22:2 ω-3**	**Docosadienoic**	0.02 ± 0.001
**C22:6 ω-3**	**Docosahexanoic**	ND
**C24:0**	**Lignoseric**	0.05 ± 0.002
**C24:1 ω-9**	**Nervonic**	0.08 ± 0.005
**Significant Oil Parameters**		
**1**	**SFA**	0.78
**2**	**MUFA**	52.15
**3**	**PUFA**	46.99
**4**	**SFA/MUFA**	0.015
**5**	**SFA/PUFA**	0.017
**6**	**MUFA/PUFA**	1.11
**7**	**SFA/MUFA/PUFA**	0.02:1.56:1.41

The results are represented as means ± SEM, where (*n* = 3).

**Table 2:** The content in mandarin seed oil of minor molecules (carotenoids, sterols, and tocopherol).

Carotenoids (mg/100 g)	Lutein	Xanthine	*β*-Carotene	Violaxanthin	*β*-Cryptoxanthin	*β,β*-Xanthophyll	Phytoene	*α*-Carotene
Mean	504.3	41.3	8.1	4.72	0.61	1.74	0.07	0.04
SEM	±2.17	±1.24	±0.9	±0.8	±0.4	±0.6	±0.03	±0.01
**Sterols** **(mg/100 g)**	**Campesterol**		**Stigmasterol**	***β*** **-Sitosterol**		***δ*** **-5 Avenasterol**	**Brassikasterol**	
Mean	31.45		8.04	294.6		5.33	0.04	
SEM	±0.94		±0.55	±3.89		±0.41	±0.002	
**Tocopherols** **(mg/100 g)**	***α*** **-Tocopherol**			***β-*** **Tocopherol**	***γ-*** **Tocopherol**			***δ*** **-Tocopherol**
mean	27.41			ND	14.85			0.09
SEM	±0.81			ND	±1.02			±0.001

The results are represented as means followed by ± SEM, where (*n* = 3).

**Table 3:** Chemical constituents of the phenolic compound contents of mandarin seeds.

Phenolic Acids	Concentrations (mg/Kg)	Flavonoids	Concentrations (mg/Kg)
Gallic acid	97.70 ± 4.57	Catechin	94.13 ± 5.81
Chlorogenic acid	ND	Catechol	133.81 ± 8.21
Protocatechuic acid	ND	Epicatechins	2.74± 0.89
*Trans*-ferulic acid	63.17 ± 1.69	Rutin trihydrate	32.42± 5.84
*Trans*-cinnamic acid	4.43 ± 1.57	Apigenin 7 glucoside	43.33 ± 2.98
Syringic acid	66.14 ± 3.58	Quercetin	73.47 ± 1.38
Caffeic acid	35.42 ± 4.66	Luteolin	ND
Ferulic acid	1.48 ± 0.34	Hesperidin	4.38 ± 0.51
*p*-Hydroxybenzoic acid	2.94 ± 0.67	Naringenin	35.13 ± 1.78
*p*-Coumaric acid	2.72 ± 0.18	Kaempferol	11.24 ± 1.34
Resveratrol	18.47 ± 2.44	Isorhamnetin	92.81 ± 2.78
Vanillic acid	0.44 ± 0.02	Chrysin	ND

The results are represented as means ± SEM, where (*n* = 3); ND: not detected

**Table 4:** Essential compound content determined for the mandarin seeds.

Compound	RI	% Volatile Fraction	Identification
Hexanal	801	0.26 ± 0.08	MS & RI
*α*-Thujene	928	0.45 ± 0.05	MS & RI
*α*-Pinene	939	6.82 ± 0.73	MS, RI & ST
Sabinene	972	0.33 ± 0.02	MS & RI
*β*-Pinene	981	4.32 ± 0.24	MS, RI & ST
*β*-Myrcene	993	1.37 ± 0.54	MS & RI
Octanal	1006	1.04 ± 0.28	MS & RI
*α*-Terpinene	1012	0.32 ± 0.05	MS, RI & ST
*β*-Phellandrene	1030	3.93 ± 0.03	MS & RI
Limonene	1033	66.05 ± 1.41	MS & RI
*γ*-Terpinene	1074	12.31 ± 0.67	MS, RI & ST
*α*-Terpinolene	1096	0.08 ± 0.01	MS & RI
Linalool	1100	0.11 ± 0.03	MS, RI & ST
Nonanal	1104	0.09 ± 0.01	MS & RI
Geranyl	1149	0.05 ± 0.02	MS & RI
Citronellal	1159	0.23 ± 0.04	MS, RI & ST
Decanal	1234	0.04 ± 0.01	MS & RI
Ethanone	1274	0.57 ± 0.05	MS & RI
Cadinene	1275	0.48 ± 0.06	MS, RI & ST
*α*-Cubebene	1345	0.09 ± 0.03	MS & RI
Isopiperitone	1473	0.59 ± 0.11	MS & RI
*α*-Sinensal	1526	0.1 ± 0.03	MS & RI
*β*-Sinensal	1675	0.4 ± 0.02	MS & RI

The results are represented as means ± SEM, where (*n* = 3). The number of essential components of the mandarin seeds was expressed in percentile ratios. MS: mass spectra; RI: retention index; ST: standard applied for identification.

## Data Availability

All the data related to this article is available at https://www.mdpi.com (accessed on 10 November 2021).

## Supplementary Materials

The following are available online. Figure S1. The reduction of the toxigenic fungal growth for the mycelia of two strains of *Aspergillus* fungi known to produce mycotoxin in their growth media.
