# Supplementary material for: Bioactive Molecules of Mandarin Seed Oils Diminish Mycotoxin and the Existence of Fungi
Source: Molecules. 2021 Nov 25;26(23):7130. doi: 10.3390/molecules26237130 (PMC8659201; doi:10.3390/molecules26237130)
Supplement: Supplementary file 1 [file molecules-26-07130-s001.zip › molecules-1479967-supplementary.pdf]

Supplementary materials for Manuscript ID : **molecules-1479967**

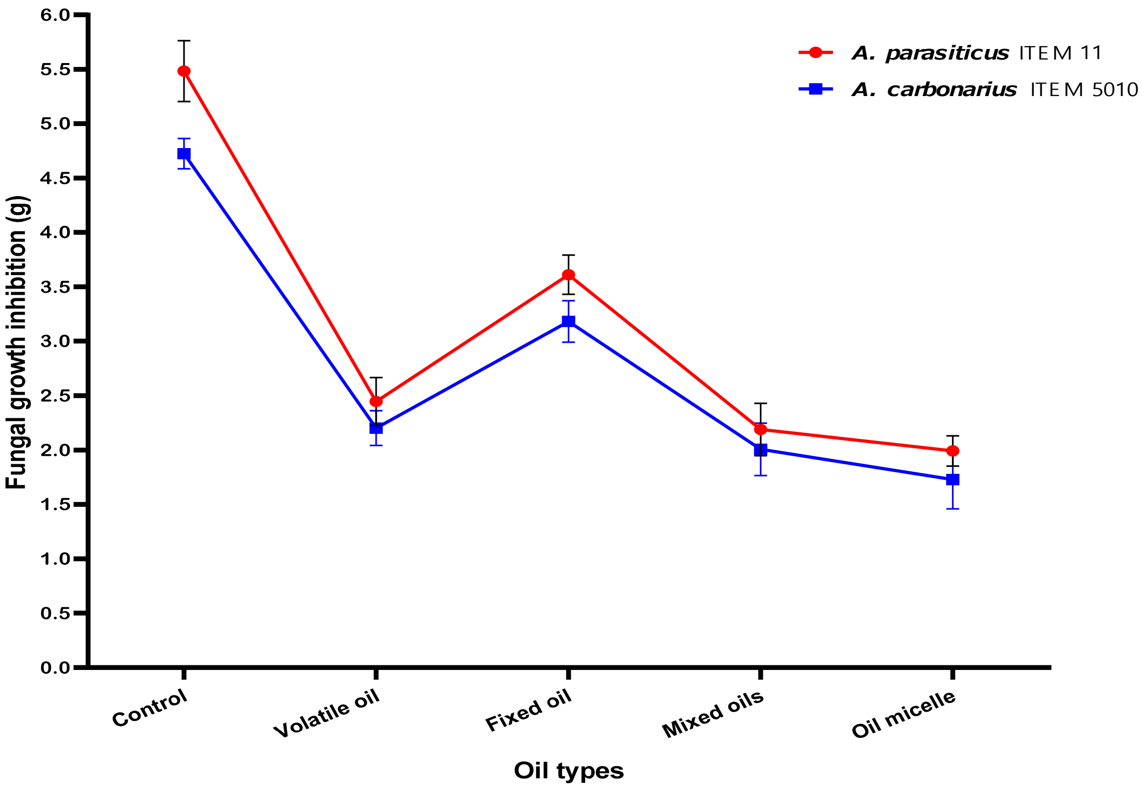

Figure S1. The reduction of the toxigenic fungal growth for the mycelia of two strains of *Aspergillus* fungi known to produce mycotoxin in their growth media.

- *A. parasiticus* ITEM 11: represents the reduction of the *Aspergillus parasiticus* strain due to the treatment by oil types.
- *A. carbonarius* ITEM 5010: represents the reduction of the *Aspergillus carbonarius* strain due to the treatment by oil types.
